# The *wow factor* as a determinant of funding for disorders of the skin

**DOI:** 10.1186/s40779-015-0040-7

**Published:** 2015-06-11

**Authors:** Terence J. Ryan

**Affiliations:** Green Templeton College, Oxford University, Oxford, OX 26 HG UK

**Keywords:** Skin disorders, Wound healing, Skin care

## Abstract

As people live beyond 100 years, there is an extended period of impaired quality of life for the increasing numbers of individuals with skin disorders. There is also a growing work force of fit elderly individuals who are able to provide low technology skin care and who can teach self-help if well instructed. The International Society of Dermatology’s sub-committee *Skin Care for All: Community Dermatology* seeks to bring together those who care for skin diseases and those who manage wounds, burns, lymphoedema and neglected tropical diseases affecting the skin for the purpose of skin care. Their focus is the repair of four functions: barrier, thermoregulation, sensory perception and communication. The curriculum includes low cost self-help and the restoration of absent skin. The care expectation is one of technical proficiency integrated with kindness and altruism. The concept is attracting wide attention but needs to develop compelling and persuasive arguments *(“wow factors”*) regarding why it should be funded. There is probably no greater *wow factor* than tracing the path of a severely injured patient from the battlefield through the course of immediate first aid by paramedics to the surgeon in the frontline tent who can almost guarantee survival. Seeing these disfigured persons winning trophies at the Olympic Games has garnered the admiration of millions of viewers.

## Background

Funding for skin disorders needs instantly and compellingly persuasive arguments in its favour. These arguments are commonly known as *wow factors.*

In the past, funds have been mostly available to reduce the number of people dying from disease. Skin disorders and their often-termed minor speciality dermatology have not been able to use mortality to aid in fundraising.

In his 2014 Harveian Oration [1], Richard Peto’s graphs of falling premature death statistics were something of a *wow factor.* It is astonishing how likely it is today that one may live to age 100, compared to not so long ago when living to half that age was not expected. In recent years, thanks especially to data collection by the Welsh School of Dermatology, quality of life statistics have greatly strengthened the case for funding skin care. Those who retire not long after middle age can expect to remain healthy until the age of at least 85. However, others must carry the burden of failing skin for longer than was previously likely before death brought relief. These lives are intolerable, and with an extended life span, their numbers are increasing the burden of skin failure. This situation justifies a completely new assessment of skin care provision and funding. The following is how a new task force *Skin Care for All: Community Dermatology* (www.skincareforall.org) [2] makes such an assessment.

### Skin care for all: community dermatology

Dermatology is not the only profession concerned with the skin, but through collaborations and education systems, it influences most of those who care for the skin. National dermatological societies worldwide formed an International League of Dermatological Societies (ILDS) that created the International Foundation for Dermatology (IFD), from which the Journal of Community Dermatology can be downloaded (www.ifd.org). The IFD is the leading voice for skin care in the developing world where there are often no national societies. The International Society of Dermatology, a member of the ILDS, is an organisation that has had individual members and a journal concerned with dermatology, ecology, geography and tropical dermatology for half a century. It has a task force, *Skin Care for All: Community Dermatology*, that was recently positioned as a sub-committee. It is concerned not only with skin diseases but also with wounds, burns, lymphoedema, and neglected tropical diseases that affect the skin, such as leprosy, leishmaniasis, onchocerciasis, podoconiosis, trachoma, yaws, and Buruli ulcer. Its focus is on skin function and failure. Dermatology has also traditionally partnered with practitioners for the control of sexually transmitted infections.

In contrast to dermatology, with its diagnostic acumen in naming thousands of diseases, skin care is concerned with the failure of four functions: barrier functions, ranging from impaired trans-epidermal water loss to absent skin; thermoregulation, which includes extremes of heat and cold; sensory impairment, which refers to itching, pain or numbness; and communication, including the “look good feel good” factor and body image.

The dermatology profession understands the widespread needs of human resources and how to meet them. However, while most dermatologists support community practice in principle, they do not want to practice outside their urban base or employ skills beyond their immense diagnostic acumen. Many enjoy the science of dermatocosmetics [3], especially in the field of skin surgery. There is no denying that overall health includes well-being and that the “look good feel good” factor contributes to this state. The focus on the cosmetic has found increasing justification, and the demand for it is increasing.

Nevertheless there are those who have taken up a more public health approach to skin disease; Table 1 lists twenty-two examples of community dermatology. This approach finds favour amongst the young; however, less enthusiastic participation is seen by those who are more established with commitments to a private practice, family and home.

**Table 1:** Global community dermatology interventions

Country	Dermatological Interventions
1) Mexico	Teams of doctors and nurses visit remote areas and provide diagnosis and management with government encouragement on a state-by-state basis [13, 30].
2) Tanzania	A Regional Dermatology Training Centre. Two-year training for allied health professions to provide skin care in 12 English-speaking African countries. An association of 260 graduates provides community dermatology over wide areas of the continent of Africa [13].
3) Mali	A health centre-based one-day course for managing the three most common skin problems: bacterial infections, fungal infections, and scabies. It improves diagnosis and eliminates expensive prescriptions for the wrong diagnosis [13, 21].
4) Nigeria	A course with a prime focus on leprosy mentored by Eric Post of the Netherlands.
5) Rome	A programme for immigrant populations lead by Professor Aldo Morrone: a hospital with adequate interpreters that manages illegality and protects both the immigrants and the local populations from untreated infections [31].
6) Puerto Rico	A programme using medical students to provide yearly skin clinics in Puerto Rico [32].
7) South Africa	A nurse-led community dermatology model with outreach visits to health centres in South Africa [4, 33].
8) Haiti	USA dermatological and surgical teams expand dermatological and dermatopathological services, educating local physicians and providing telemedicine and medical supplies, led by Morrison B, Vega A, and Vega (E-mail: theskinclinic.haiti@gmail.com) and John Macdonald, Hospital Bernard Mevs Project Medishare, Port-au-Prince (E-mail: Trappermac@AOL.com).
9) UK	Dermoscopy courses for family practitioners and other health professionals to detect skin malignancy. South Coast Dermoscopy Associates Integrated skin lesion recognition and dermoscopy run by Stephen Hayes (E-mail: hayes373@btinternet.com).
10) UK	The Welsh School of Medicine provides family practitioner training globally through distance learning courses: MSC in Clinical Dermatology, Diploma in practical Dermatology and Introduction to Dermoscopy (www.dermatology.org.uk).
11) Ethiopia	Focus on foot disorders (e.g., podoconiosis) for one million shoeless rural agricultural workers in irritant soil (www.podo.org). It provides microfinances for women who are concordant with washing and foot care. Recently added to the neglected tropical diseases [14].
12) UK	Self-help at a low cost: a programme of patient empowerment to develop clean water washing, oiling, skin protection, mobility, not smoking, elevation of legs, gardening (nutrition) for health [34] and to care for disfigurement.
13) India	Three-month courses on community dermatology (three modules, three workshops and three evaluations, as well as courses for community nurse management (E-mail: neelimas1@gmail.com).
14) Kerala	A programme for elephantiasis, vitiligo, wounds, and psoriasis, integrating Ayurveda, yoga and homeopathy with biomedicine and incorporating patient participation and environmental improvements [6].
15) Nepal	Dermatologists providing outreach programmes for skin care for some of the most remote and seasonally climate-affected peoples of the world [35, 36].
16) Patagonia	Clinical teaching and health programme aimed at rural communities run by an Argentinian dermatologist and helped by volunteers from dermatology departments throughout Argentina [13].
17) Argentina	One million immigrants from Europe: a campaign to manage the high prevalence of skin cancer by Gastón Galimberti Coordinador del Centro de Cancer de Piel.
18) Cambodia	A programme designed to teach general medical officers dermatology at a basic level appropriate to the regional needs combined with a new programme to re-introduce specialist training in dermatology [13].
19) Africa	The building of diagnostic capacity in dermatopathology in resource-poor areas of East Sub-Saharan Africa (funded by the International Foundation for Dermatology (IFD) and European Academy of Dermatology and Venereology (EADV) and managed by Dr Helmut Bertramelli, [37]).
20) China	A model led by burn units cooperating with trauma focusing on absent skin of all aetiologies to provide public education and, eventually, it is hoped, skin care for 56 ethnic groups especially in West China. Linked to this will be courses on rehabilitation planned at Oxford for young Chinese graduates sponsored by the burn and trauma care industry; a partnership between Oxford Brookes University and The Second Affiliated Hospital of Anhui University of Traditional Chinese Medicine. Details from TJ Ryan (E-mail: userry282@aol.com).
21) India	The Bombay Leprosy Project (www.bombayleprosyproject.org) [38] is well established and has many years of experience managing leprosy in an agrarian to urban migration under very resource-poor conditions.
22) Australia	Health interventions by medical students using their elective periods and young doctors and nurses for indigenous populations, both for continental Aboriginals and for Pacific islanders in Fiji to control scabies [16, 19].

### Tissue viability of wounds, burns, lymphoedema and neglected tropical diseases

Over the last century, the Department of Dermatology at Oxford has focused on wound healing, helping to found organisations such as *the European Tissue Repair Society, The European Pressure Ulcer Advisory Panel, The British Lymphology Interest Group, and The International Skin Care Nursing Group* [4] (affiliated with the International Council of Nursing), concerned with wounds and especially with leg ulcers, diabetic feet and pressure ulcers. There is ample confirmation that tissue viability and research into skin care are an appropriate collaboration.

Of special interest have been contributions in the two nations whose populations exceed one billion. The Institute of Applied Dermatology in Kerala, India has reached out to care for the many millions of individuals who are disfigured and often bedridden by the swollen legs of lymphatic filariasis. This situation underscores the value of not ignoring the fact that traditional health practice or complimentary or alternative medicine are in many countries the first health systems to be called upon. Thus, in India, Ayurveda and homeopathy provide a large human resource contributing to well-being and amenable to important improvements in knowledge [5]. Integrative medicine finds an effective model in outreach to rural villages [6].

A few decades ago, military medicine in China sent young doctors to the Department of Dermatology at Oxford to learn wound healing skills. Chinese dermatologists have not shown enthusiasm for community dermatology in spite of a history that includes significant pioneers in this field, such as Ma Hai-De [7], who founded the Institute of Dermatology, Nanjing, and planned the control of syphilis, tinea capitis and leprosy. He would be horrified at the recrudescence of syphilis in China. However, in today’s military, medically trained generals trained in dermatological approaches to wound healing are heading the Chinese National Trauma Association and the Chinese National Burns Society in China. Interestingly, they are also willing to teach public health responses to diabetic feet and pressure ulcers. They may also take *narrowing the knowledge gap* [8] beyond the environs of Shanghai and Beijing to include 56 ethnic groups in the West and perhaps a response to the Chinese obesity epidemic and accompanying increase in diabetes.

The author has made many visits to China, initially as the President of World Microcirculation, through interest in the blood supply of the skin and studies of Chinese traditional medicine led by Professor Xiu Rui-Juan at the Institute of Microcirculation in Beijing. Later visits were through the encouragement of Ma Hai-De [7] and the experience of visiting leprosy villages where there was clearly a need for improved skills in managing absent skin. It became clear that experts in burn surgery were talking to the Chinese government about accident prevention. They were teaching first aid to the Chinese public and becoming leaders in the field of tissue regeneration. They had experience managing severe disfigurement and recognised the value of integrating traditional Chinese medicine into biomedical practice where such an integration could be shown to be an advantage. In the context of skin care for all, burn publications and lectures at society gatherings have focused on other causes of absent skin. The World Health Organization hosted the World Alliance for Wound & Lymphedema Care (WAWLC; www.wawlc.org) and it is now recognised that the Chinese Military burn surgeons should add a discussion of burn management that can be simultaneously offered as interventions for diabetic, venous or pressure ulcers and that these should be added in 2015 to the second edition of *Wounds and Lymphedema Management* [9] published by the World Health Organization. It will also be necessary to attend to the needs of the ageing population in one-child families with children who work and are unable to prevent and manage pressure ulcers in their elderly relatives.

This is one of many examples of how those who care for the skin must focus on the multiple causes of skin failure. However, it is also an example of how Chinese burn surgery can contribute to *Skin Care for All: Community Dermatology*.

One of the benefits of people living longer is that there is a potential army of otherwise unemployed 65- to 80-year-old caregivers in good health. They can easily utilise the non-invasive demands of skin care that are achievable using low technology and with low cost. They are the grandmothers of the community. Their acquired wisdom and their role in grooming the young are as ancient as *Homo sapiens.*

### What is community dermatology?

Many dermatologists, including Roderick Hay, Gurmanhan Singh, Roberto Estrado, and Aldo Morrone have contributed to the definition of community dermatology. Community dermatology embraces common skin diseases. It is inclusive of wounds and lymphoedema but has long neglected tropical diseases such as leprosy as a major interest. It requires data on prevalence and human resources and trains community-based workers to manage common skin problems. Its interventions are low-cost and address the needs of those with few resources, although not exclusively. These populations may be isolated, peri-urban, or mobile, often against a threatening background such as strife or climate change.

Community dermatology [10] is a concept that extends the focus of care delivery from the individual patient who comes to see the doctor about a skin problem to a proactive intervention to improve the care of skin and diseases that present with skin signs in the broader communities in which they live. In this way, community dermatology is applicable in the richer countries as well as the poorer. For instance, holding pigmented mole clinics or promoting sun safe practices are examples of community dermatology. Essential to this is the idea that one uses all means of achieving this goal, and therefore, those who can contribute will vary from situation to situation, but programmes that use local health care workers, those that use traditional practices and those that involve other groups such as burn care experts are all manifestations of the same basic principle that to achieve the best results, teams are usually multidisciplinary and sensitive to local practices and needs.

The public health-based task force for *Skin Care for All* [11] manages *skin failure,* which includes the loss of intact barrier function, thermoregulation, and sensory defects giving rise to itching, pain, or numbness. It also addresses skin-related communication, such as colour prejudice, love at first sight, or the look good feel good factor. Stigmatisation and being unwelcome makes participation all the more difficult. The website www.skincareforall.org shows a capacity for benefit and advertises considerable achievements in public health fields.

Several branches of community dermatology choose to focus on common problems such as bacterial, fungal or parasitic infections, eczema, pigmentation disorders or the prevention and early diagnosis of skin cancer. Neglected tropical diseases often present predominantly with skin impairment [12]. Locally high prevalence has required a special focus on albinism in Tanzania [13], podoconiosis in Ethiopia [14], and lymphatic filariasis in Kerala, India [6]. In addition, improved survival from severe burns in China due to improved technology has been demonstrated [15].

Since the elimination of smallpox, skin conditions severely affecting whole communities have become less of a problem; scabies, however, justifies a community approach [16, 17]. Special emphasis on community housing conditions in care homes and prisons is also necessary [18]. Community dermatology enlisting the help of medical students on elective rotations has shown effective management in island communities such as Fiji [19].

It should be noted that this approach emphasising interventions for skin care through managing only four functions of the skin acts as a gateway that excludes dermatology’s great expertise in recognising, naming and managing thousands of rare diseases. Dermatologists’ training is lifelong in this respect, and hopefully, every region will have at least one centre with such expertise to serve as a referral centre for the more uncommon conditions. Community dermatology was practiced by Gurmohan Singh and Kaur in tribal communities in Bihar decades before it was re-conceived in Ethiopia in 2007 by Shyam Verma [2], a dermatologist from Gujarat, India and editor of the *Indian Journal of Dermatology (online*). In 2009, the task force *Skin Care for All: Community Dermatology* chaired by Terence Ryan was launched in Berlin by the International Society of Dermatology [2].

Many who were concerned by the increasing burden of skin disease especially in areas of poverty, called some twenty years ago for a programme to be prepared for the ILDS with suggestions for *Healthy Skin For All* [20]. Such a programme was prepared with a grant from the Education and Science divisions of the United Nations Organization for Education, Science and Culture (UNESCO). It was named UNIDERM.

UNIDERM was a five-year plan for the International League of Dermatological Societies prepared for the first five years (1989–1994) of the International Foundation for Dermatology. The programme was later named *Healthy Skin for All* [21]*.* WHO responded by letter in 1992: “We identify completely with its aims and objectives. Indeed, we could not fail to be impressed by such a comprehensive statement of commitment to the needs of the developing world by a globally representative medical body. If its ambitions are realized, its impact will clearly be exemplary.”

### The programme of *Skin Care for All:* community dermatology

The ideas behind the suggested programme are achievable. There are so far two main themes under development: 1) self-help at low cost and 2) the healing of absent skin. In addition, care for lymphoedema is also included (Table 2).

**Table 2:** Main themes of the current programme of *Skin Care for All*

Main themes	Requirements
Self-help at a low cost^(a)^	a) Cleaned with water fit for drinking using all available cleansing technology [39]
	b) Oiled [40]
	c) Kept moving [41]
	d) Positioned to reduce gravitational overload
	e) Protected from fire and scalding, excess sunlight or cold, female genital mutilation, and offloaded from excess pressure
	f) Adequately clothed and fitted with footwear; provided with mosquito nets, condoms
	g) Provided with *Gardens for Health* [34]
	h) Stop tobacco smoking [42, 43]
	i) For disfigurement, allow nature to repair but ensure added human kindness and care
The healing of absent skin^(b)^	a) Remove causes, such as fire, land mines, dangerous driving, mycobacterium ulcerans, etc.
	b) Treat systemic diseases such as anaemia, diabetes, HIV/AIDS
	c) Remove space-occupying material, such as foreign bodies, pus, haematoma, dead tissue, etc.
	d) Keep moist but do not macerate
	e) Manage oedema and care for surrounding skin to prevent the repair mechanisms of neglected skin from stealing the blood supply needed for the wound
Care for Lymphoedema^(c)^	a) Keep moving by massage, yoga, full range joint movement, deep breathing
	b) Reduce venous overload by elevation and ankle movements
	c) Reduce inflammation from bacteria, irritants, allergens, etc.

^(a)^ Self-help at low cost expects Ministries of Health to provide essential drugs for bacterial, fungal and parasitic infections, as well as for eczema and pigmentation disorders

^(b)^ Absent skin from such causes as burns, wounds, venous ulcers, diabetic foot ulcers, pressure ulcers, sub-epidermal blisters, toxic necrolysis, Buruli ulcers, etc. [9, 43]

^(c)^ care of lymphoedema [44, 45]

### The meaning of skin care

*Skin Care for All* supersedes *Healthy Skin for All,* as it has a greater chance of achievability. To care about or for care and to give or receive care involve both technology and attitude. They require the competence, responsibility and attentiveness of the highest level of professionalism as well as responsiveness and participation of the vulnerable. It is the opposite of “I don’t care.” It is not a paternalistic model but rather it expects altruism and kindness.

The Canadian physician Sir William Osler, who died 100 years ago at 13 Norham Gardens (Norham Gardens is now the Oxford office of Terence Ryan, where he acts as curator of the memory of Sir William Osler for its owner Green Templeton College), Oxford, will be celebrated in 2019 largely because his views on caring and professionalism are considered to be well expressed and still relevant today. His medicinal resources were few, but his capacity to benefit through the expression of his personality continues to be appreciated [22]. It was in 1915, exactly one hundred years ago, that he prepared the Chinese edition of his *Principles and Practice of Medicine* from Oxford*.*

During visits to China, the author has observed the advanced technology that preserves the lives of persons with 100 % burns, some of whom are blind and some of whom have suffered a loss of limb. This requires the highest care technology as well as the kindness without which a long life would be intolerable. Publications from China emphasise both technical skills and the support necessary to preserve the will to live [23].

Azulay-Abulafia *et al.* [24] emphasised that the community approach teaches us to love our profession.

### Implications of being a part of public health

We join Universal Health Coverage to ensure that all people are able to access the “quality” health services they “need” without suffering financial hardship. There is concern about avoidable waste, clean water systems, health centres without toilets, gardens for health, care of mosquito nets, care of neonatal umbilical cords, care of circumcision wounds and avoidance of female genital mutilation. All individuals should be able to access safe water and sanitation, electricity, connection to information technology and primary health care, and be protected from hazards.

The threat of climate change is a hot topic for dermatology [25] and protection against its hazards is clearly something to which community dermatology and its collaborators can contribute.

All should have an understanding of the information needs of health care providers in low-income countries and should seek to ensure that evidence based medicine is culturally acceptable, available and affordable as well as environmentally compatible. It should be reproducible and generalisable, and ones’ patients should resemble published trial subjects when following recommendations from Cochrane reviews.

Amongst tribal and aboriginal communities, perceived relationships between man and the environment as well as oral traditions and the wisdom of traditional health should be respected. Traditional health practitioners are a huge potential workforce, both sustainable and culturally competent. They need to be educated for integration with biomedicine, and all systems need to be made safe [26]. Every older child and every fit elder can be a health provider to his or her own family.

### Objectives for minimal care of the skin: global, self-help/patient empowerment at a low cost

The above aims are for everyone who cares for the skin, whether doctor or nurse, dermatological disease specialists, wound lymphoedema or burn therapists and all those caring for neglected tropical diseases associated with impairment of skin function. They should be used as an education package, and no single profession should regard them as written solely for them. Indeed every profession caring for the skin should hand share them and supervise their distribution to all in need. The mobile/smart phone can be reliably preloaded; currently 25 % of websites are viewed this way. The web site of World Vision provides valuable guiding principles for community health workers. Funding is needed to narrow the knowledge gap, as expressed in the following WHO document: [7].

**Fig. 1:**
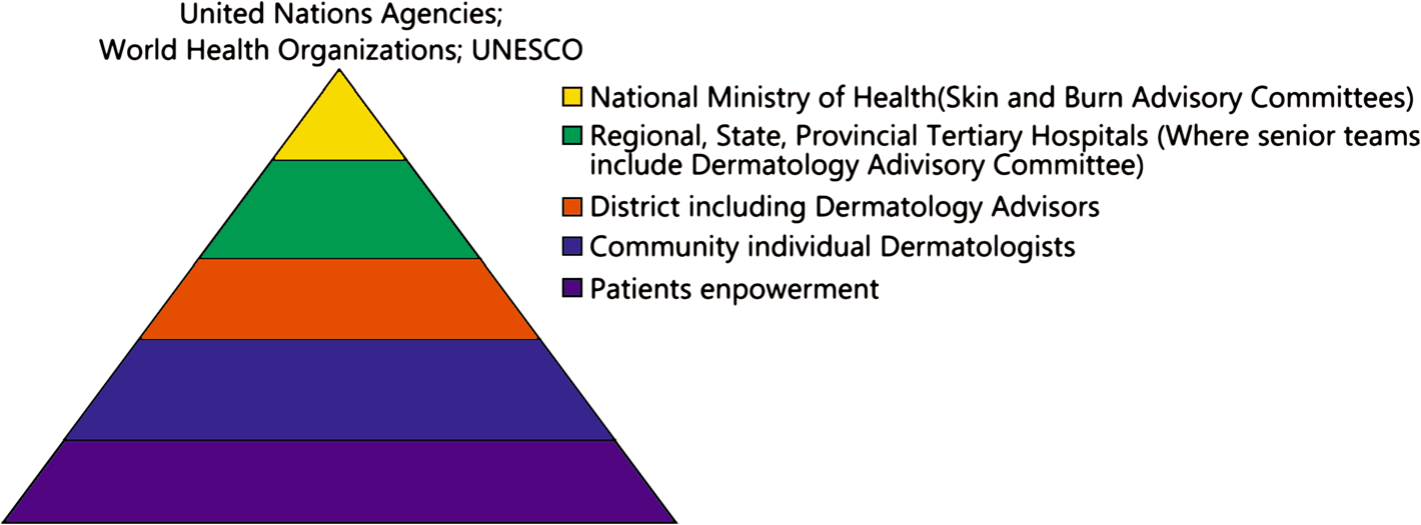
The pyramid of skin care

“To better inform and thus empower individuals and the public to make the right decisions regarding their health and wellbeing, influence public health policy-making and decision making by positioning your profession in the upper reaches of the pyramid of health (Fig. 1) where besides educating the community there is an essential role as educator of those who govern health policy. To advance the frontiers of knowledge to develop products and tools for the promotion, maintenance, protection and restoration of health.”

Until now, skin care has had insufficient appeal when recruiting human resources. Indeed its failure with severe disfigurement even repels some observers. A search for *wow factors* is recommended. There are also organisations not directly concerned with health that can be recruited. Thus, the author has recruited the London College of Fashion and its International body of students to help to prepare a website www.skincareforall.org and documents that show how disfigurement can be beautiful and how *better lives can be ensured* with the aid of clothing, cosmetics and jewellery.

Over the long term, all must seek funding for salaries and security of tenure for the currently non-existent administration and staff of community dermatology if they are to answer the call by Anders Seim [27] in 2005, that it is “*time for an additional paradigm? the community-based catalyst approach to public health.”*

This paradigm must take into account the long life of the severely disfigured and the increasing numbers of persons who have skin failure now frequently addressed by transplanted tissues, including the entire face [28], hairless and unable to sweat, itching, painless or numb, and though with a less distorted body image, hardly the confident nude that Ryan described in 1987 as the desirable end point of skin care [29].

### Military medicine shows the way

In a year that has recalled the great war of 1914–1918 and Médecins Sans Frontières (MSF) has performed wonders in the field of conflict, much attention has been given to the extraordinary route taken by large numbers of the severely wounded from resuscitation by the paramedic to the almost guaranteed survival after surgery in the front line tent, ending up as a trophy winner at the Olympic Games and *wowing* millions of TV viewers worldwide. Military surgeons have found a civilian population that is increasingly requesting their help as a result of injuries from land mines or earthquakes or climate change disasters. Apart from first aid at the onset of a threat of skin failure, community dermatology has much to offer in maintaining the integrity of the skin in the years after an injury, for example, the skin of an amputation stump. One hundred years ago, long-term survival after severe injury was unlikely, while today, survival is an almost 100 % possibility when a patient is alive at the time of presentation to the paramedic. The numbers requiring skincare are increasing, and the burden is no longer small or likely to be foreshortened by death. It is an outcome that requires a public health response. There is much to rethink, and Skin Care for All must contribute from birth to the closing moments of palliative care. Hippocrates exhorted “to cure sometimes, to relieve often, to comfort always.” It is necessary for every professional, including the military, with an ever-increasing capacity to mend, to practice palliative care as a comfort measure.

In 1959 the Director General of the British *RoyalArmy Medical Corps* initiated a civilian programme and directed TerenceRyan as *officer in charge* of Dermatology and Ear Nose and Throat surgery at Colchester Military Hospital (which received all the evacuations from the *British Army of the Rhine,)* to pioneer the programme. Throughout his career he has supported a civilian assistance role for armed services.

## Conclusions

Skin care is a global need and it can be delivered by all who care for the skin. There are no funds to support those in Dermatology who wish to make a career in community dermatology. This is unfortunate because there are a small number of dermatologists who have a passion for public health and can join many other disciplines who can contribute to restoring skin function. During recent years the concept of Skin care for all: Community Dermatology has acquired an active following especially in India from Dermatologists and in China the public health interests of military burns surgeons have broadened into education about the diabetic foot and pressure ulcers. By including wounds, burns, lymphoedema and many Neglected Tropical Diseases under the heading of Community Dermatology it is possible to envisage a new era of skin care as a Public Health discipline. It will provide a potential of participation and inclusion for the many who currently suffer from failure of the skin as an organ, are often unwelcome, they are at risk due to the absence of an effective barrier to a threatening environment and suffering from itch, pain or numbness.
